# The first occurrence of the enigmatic archosauriform *Crosbysaurus* Heckert 2004 from the Chinle Formation of southern Utah

**DOI:** 10.7717/peerj.905

**Published:** 2015-04-21

**Authors:** Robert J. Gay, Isabella St. Aude

**Affiliations:** Science Department, Mission Heights Preparatory High School, Casa Grande, AZ, USA

**Keywords:** Crosbysaurus, Chinle Formation, Chinle, Utah, Comb Ridge, New occurance, New record, Triassic, Late Triassic, Archosaur

## Abstract

Originally identified as an ornithischian dinosaur, *Crosbysaurus harrisae* has been found in New Mexico, Arizona, and its type locality in Texas, as well as in North Carolina. The genus has been reassessed by other workers in light of reinterpretations about the postcrania of another putative Triassic ornithischian, *Revueltosaurus*. The understanding of Triassic dental faunas has become more complicated by the extreme convergence between pseudosuchian archosaurs and ornithischian dinosaur dental morphologies. We report here on a new specimen of *Crosbysaurus* (MNA V10666) from the Chinle Formation at Comb Ridge in southeastern Utah. This new specimen is assigned to *Crosbysaurus* sp. on the basis of the unique compound posterior denticles, labiolingual width, and curvature. While MNA V10666 does not help resolve the affinities of *Crosbysaurus,* it does represent the extension of the geographic range of this taxon for approximately 250 kilometers. This is the first record of the genus *Crosbysaurus* in Utah and as such it represents the northernmost known record of this taxon. This indicates that *Crosbysaurus* was not limited to the southern area of the Chinle/Dockum deposition but instead was widespread across the Late Triassic paleoriver systems of western Pangea. The reported specimen was found in close association with a typical Late Triassic Chinle fauna, including phytosaurs, metoposaurs, and dinosauromorphs.

## Introduction

*Crosbysaurus harrisae* was first described by Heckert (2004) under the assumption that it, like the better-known *Revueltosaurus*, was an ornithischian dinosaur. Several purported ornithischian taxa were named upon the discovery of isolated teeth, leading several authors to suggest that herbivorous dinosaurs were widespread across Pangea (Hunt & Lucas, 1994; Heckert, 2002; Heckert, 2004; Heckert, 2005). This scenario contrasted sharply with previous views on ornithischian diversity and stood in sharp contrast with the non-dental fossil record of ornithischians outside of the southern hemisphere.

This interpretation based on isolated teeth was challenged by Parker et al. (2005) with the discovery of the postcrania of *Revueltosaurus* from the Petrified Forest of Arizona. This contribution, not only shed light on how *Revueltosaurus* was seen but it called into question the systematics of all purported ornithischian dinosaurs from North America (Irmis et al., 2007; see also Nesbitt, Irmis & Parker, 2007). Without any supporting of skeletal remains it was no longer unambiguous to assign “fabrosaur”-like teeth to any known dinosaur clade. While *Revueltosaurus* is now known from cranial and postcranial elements, other supposed ornithischians known from only isolated teeth, such as *Tecovasaurus murrayi* and *Crosbysaurus harrisae,* can only be identified as being archosauriforms of uncertain affinity. While some authors have suggested that ornithischians were present in the Late Triassic of North America (Heckert, 2005) virtually all authors are in agreement that *Crosbysaurus* cannot be diagnosed beyond an indeterminate archosauriform and new data suggest that it may be instead an non-archosauriform archosauromorph based on similar dental features found in the archosauromorph *Azendohsaurus* (Flynn et al., 2010). While this new record does not clarify the systematic affiliations of *Crosbysaurus* it does significantly extend its range. Previous reports of *Crosbysaurus* in the southwestern United States have been limited to Texas (the type locality), New Mexico, and Arizona (Heckert, 2004). Comb Ridge in southeastern Utah is approximately 245 km away from the closest reported *Crosbysaurus* remains in the Chinle Formation of Arizona.

In May of 2014 ten students from Mission Heights Preparatory High School traveled to southeastern Utah. Students prospected the Chinle Formation exposed at Comb Ridge and opened a test pit at a possible metoposaurid temnospondyl site discovered by the first author in March of the same year. The second author, accompanied by another student, discovered a rich locality to the south of the metoposaur site. The second author and another student named this rich microsite “The Hills Have Teeth.” While combing the ground near the base of The Hills Have Teeth locality (MNA Locality 1724) the second author discovered an unusual partial tooth, MNA V10666, to the west-southwest of the main outcrop. This second locality has been designated MNA Locality 1725. The students brought this tooth to the attention of the first author and it is described here.

## Materials and Methods

Standard paleontological hand tools (ice picks, dental tools, natural-bristle brushes, plastic bags) were used to collect MNA V10666. Geographic locality data were recorded via BackCountry Navigator Android Application running on a Samsung Galaxy S4. MNA V10666 was collected under Bureau of Land Management paleontology permit UT14-001S issued to the first author and are curated at the MNA. The stratigraphic section was measured using a standard 1.8 m Jacob Staff and a Brunton Pocket Transit. Figures and line drawings were produced using GIMP 2.8.4. Photos used for figures were obtained using an Olympus E-500 DSLR camera. Specimen measurements were obtained using Craftsman (model 40257) metal sliding calipers with a precision of 0.05 mm.

## Systematic Paleontology

Diapsida Osborn, 1903

Archosauromorpha Von Huene, 1946

?Archosauriformes Gauthier, 1986

*Crosbysaurus* Heckert, 2004

### Type material

The type material of *Crosbysaurus* comes from Crosby County, Texas, and consists of a single tooth (NMMNH P-34200). Several other teeth were collected from the same locality and are paratype specimens: NMMNH P-34201, NMMNH P-34319, NMMNH P-34320, NMMNH P-34260, NMMNH P-34261, NMMNH P-34262, NMMNH P-34393, NMMNH P-34394, and NMMNH P-34397 (Heckert, 2004). All teeth illustrated by Heckert (2004; Figs. 51–54) are labiolingually compressed with compound denticles on the distal edge. In labial view the holotype and paratypes possess a conical and short outline. The figured teeth have a slight labial bulge but are otherwise symmetrical in occlusal view.

### Referred material

MNA V10666, a single shed tooth crown.

### Locality and horizon

**Figure 1:**
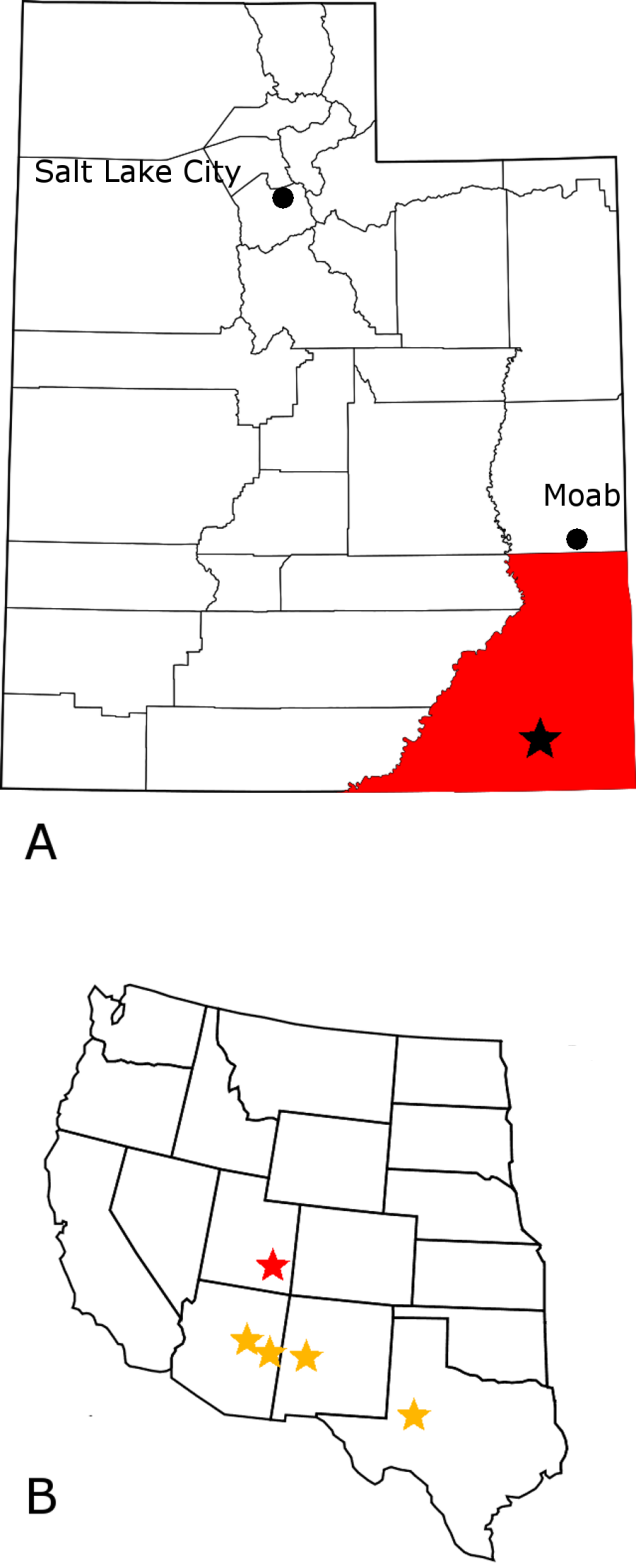
*Crosbysaurus* localities in the western United States. Map showing (A) the location of MNA Locality 1725, The Hills Have Teeth (starred), San Juan County (highlighted), Utah, USA; (B) the location of MNA locality 1725 (red) relative to previously reported *Crosbysaurus* sites (yellow) in the western United States.

**Figure 2:**
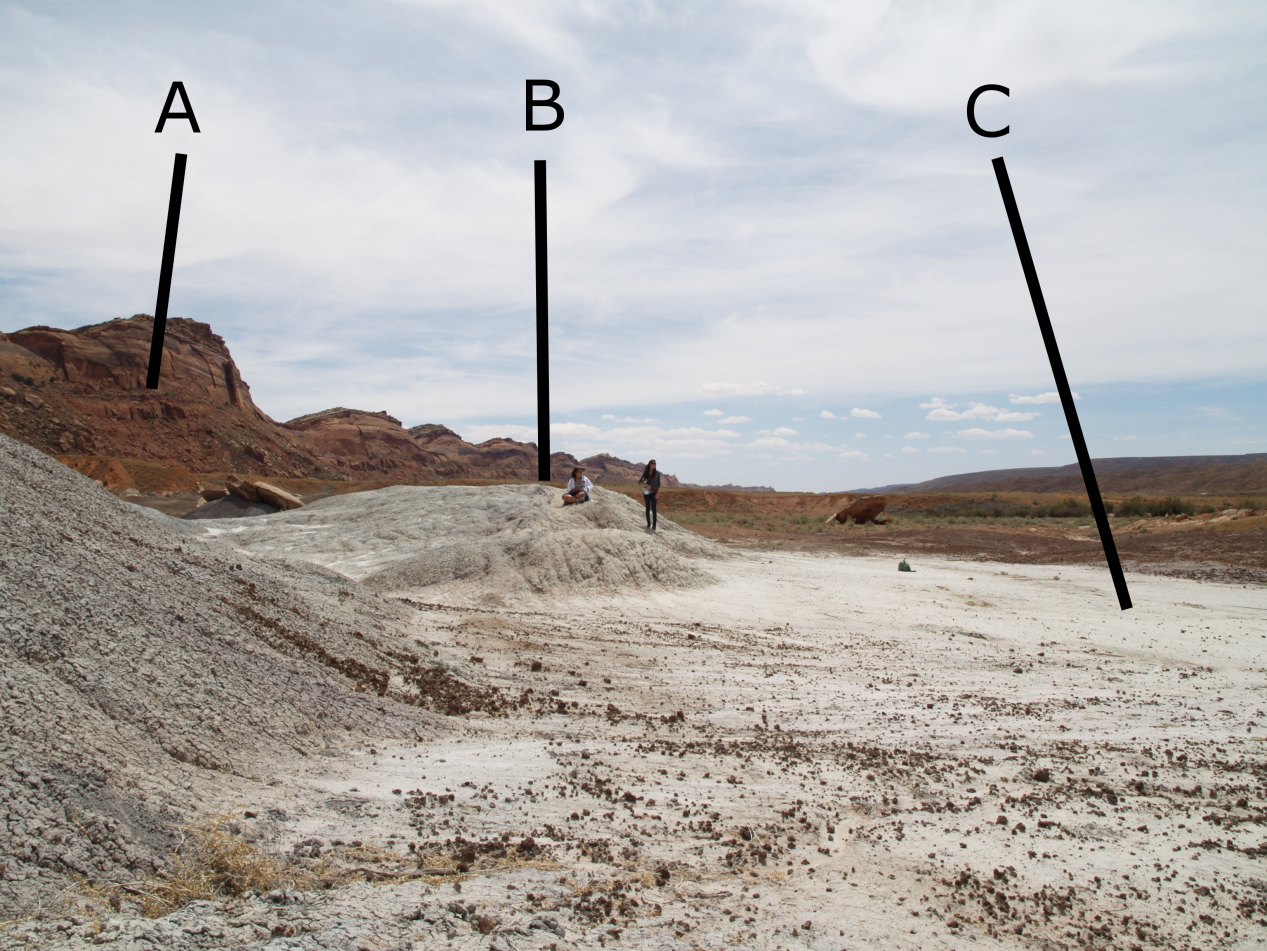
The Hills Have Teeth locality photo. MNA Locality 1725, showing relative stratigraphic position of MNA V10666 to the surrounding sediments. (A) Top of the Chinle Formation at Comb Ridge; (B) The second author sitting at The Hills Have Teeth (MNA Locality 1724); (C) location where MNA V10666 was discovered.

MNA 1725 is located in San Juan County, Utah (Fig. 1). The exact coordinates remain on file at the Museum of Northern Arizona. This locality, named The Hills Have Teeth, produced numerous partial and complete phytosaur and metoposaur teeth along with several dinosaur or dinosauromorph teeth. MNA V10666 was found approximately four meters west-southwest of The Hills Have Teeth outcrop as surface float, six meters above the Moenkopi Formation and very likely has originated at The Hills Have Teeth (Fig. 2). This is corroborated by the presence of phytosaur tooth fragments found close (within 10 cm) to MNA V10666 which the second author was able to connect with fragments collected at the main deposit at The Hills Have Teeth. The lack of recent damage to the fine mesial denticles further supports the hypothesis that MNA V10666 was not transported post-exposure very far from where it was discovered.

This area has not been mapped in detail but the upper portion of the Chinle Formation have been reported to correlate to the Petrified Forest Member by Bennett (1955), the Rock Point Member (Lucas et al., 1997), the Owl Rock Member (Molina-Garza, Geissman & Lucas, 2003), and the Church Rock Member (Martz, Irmis & Milner, 2014). Further work by the authors and others is ongoing and the relationships between the beds at Comb Ridge and other exposures of the Chinle Formation will be clarified in the near future. None-the-less, it is clear that MNA V10666 was originally deposited near the base of the Chinle Formation as part of the earliest fauna recorded in the Comb Ridge area.

The precise fossil-bearing horizon of MNA V10666 has not been identified but the nearby Hills Have Teeth outcrop consists of fine white to grey mudstones and siltstones here interpreted as floodplain deposits. The uppermost horizon at The Hills Have Teeth is nine meters above the Moenkopi Formation, giving an upper limit to the stratigraphic position of MNA V10666. Based on the nearby fossil deposits, it is likely that MNA V10666 originated from these floodplain deposits as well.

**Figure 3:**
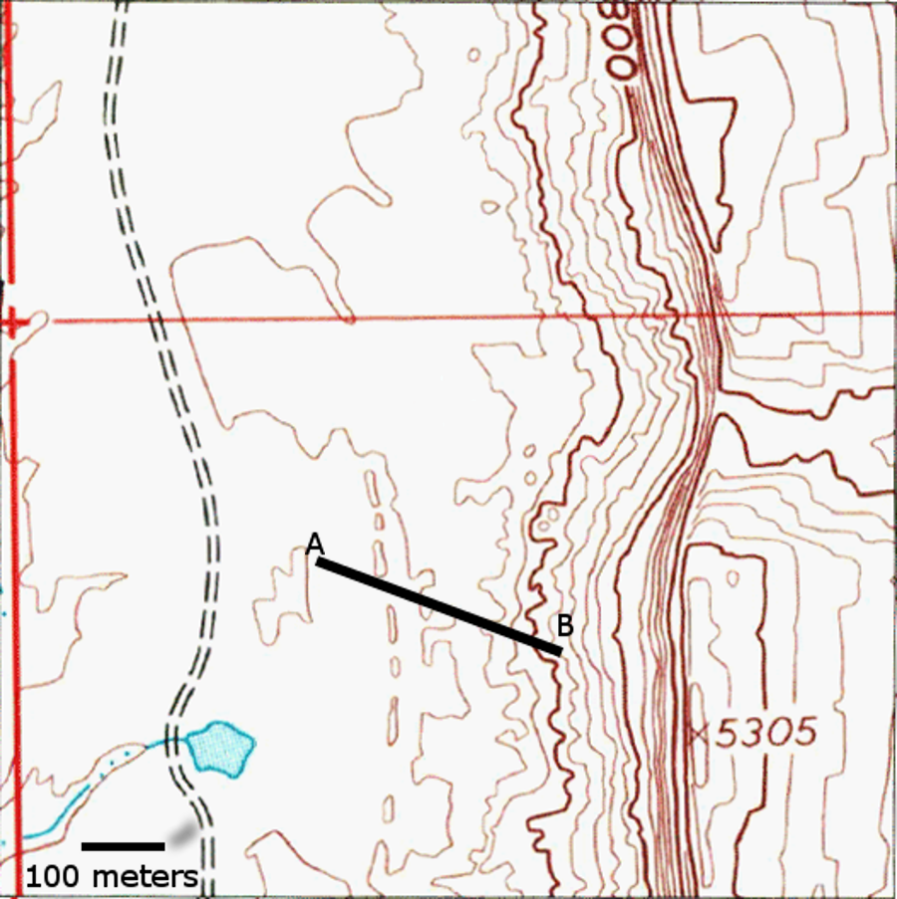
Location of measured stratigraphic section in Fig. 4. North is to the top of the figure. Topographic map data from USGS. Scale bar = 100 m.

**Figure 4:**
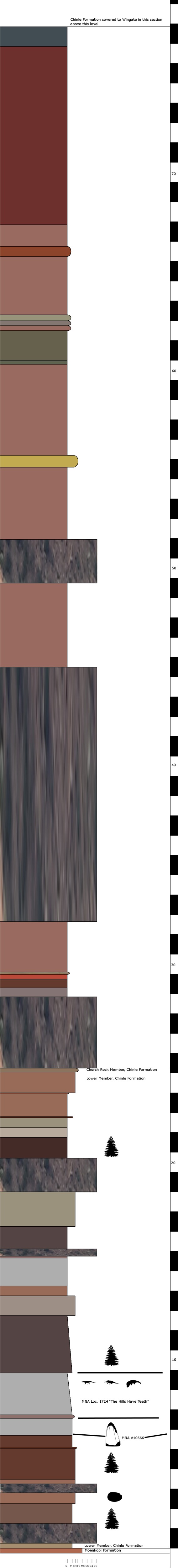
Stratigraphic column of the Chinle Formation at the Hills Have Teeth. Measured section through the Chinle Formation exposed at The Hills Have Teeth locality. MNA V10666 is represented here by an outline diagram. Scale bar = 1 m. Organism silhouettes from Phylopic by Dmitry Bogdanov, Nobu Tamura, Scott Hartman, and Robert Gay.

In order to test this stratigraphic hypothesis, the first author measured a stratigraphic section through The Hills Have Teeth section (Fig. 3). The Chinle Formation exposed at Comb Ridge has a thickness of 76.8 m with an estimated 77 m of section covered by colluvium up to the contact with the Wingate Formation. The strike and dip of the Chinle Formation at this section is 024°/114°E. This remained consistent throughout the section measured at The Hills Have Teeth. The lower member has a thickness of 24.2 m and contains multiple horizons of bentonite clays. The lower portion of the section is fossiliferous with abundant petrified wood. Silicified wood remains become rare at the upper part of the section with no wood present above the 21 m level. Significant vertebrate remains are so far constrained to the lower nine meters of the formation, though indeterminate vertebrate fragments were found as high as 12 m above the Moenkopi Formation. The vast majority of vertebrate remains (>95%) in this section come from light grey bentonitic mud-grading-to-shale, forming The Hills Have Teeth outcrop. No *in situ* fossil remains have been found below The Hills Have Teeth section. The unionid bivalve found three meters from the base of the Chinle Formation, below MNA Locality 1725, was recovered on the surface (Fig. 4). Similarly, the phytosaur and temnospondyl teeth recovered from the alluvial fan below The Hills Have Teeth outcrop, alongside MNA V10666 were collected as float.

In general, the lower member of the Chinle Formation appears grey to dark blue on the surface. Bentonite is common throughout the member. Paleosols are rare. Caliche development is common with seven distinct zones of caliche present between the base of the Chinle Formation and the contact with the Church Rock Member. A limey sand, possibly representing a short period of standing water, is present as a discontinuous bench at the base of The Hills Have Teeth outcrop, 12.6 cm thick.

The contact between the lower member and the Church Rock Member of the Chinle Formation occurs at caliche-rich reddish-brown silt. Generally, the Church Rock Member appears more reddish or pinkish on the surface. More of this member is covered by colluvial and alluvial deposits in this section, so the stratigraphy is less well defined above the contact with the lower member. The Church Rock Member appears more homogeneous than the lower member. Most of the exposure consists of weak red mottled white mudstones through the majority of the section. This mottling may indicate reduction mottling and becomes more common towards the top of the measured section. Six distinct caliche beds were measured in the section, including the caliche present in the contact with the lower member. Other caliches occur in the upper 20 m of the measured section. In contrast with the lower member, the Church Rock appears to lack bentonite in this locality, in line with other exposures of the Church Rock in Utah and across the Colorado Plateau (Martz, Irmis & Milner, 2014). No conglomerate beds exist in the measured section in contrast to those seen further northeast (Martz, Irmis & Milner, 2014) though conglomeritic lenses occur further north at Comb Ridge. While both vertebrate and plant fossils are common in the Church Rock Member in Lisbon Valley and to the northwest (Martz, Irmis & Milner, 2014), no fossil remains were recovered above the lower member in this section of Comb Ridge. Indeed few fossils have yet been found in the Church Rock Member at Comb Ridge. While this may reflect the actual preservation of fossil material in the Church Rock it is also likely influenced by the amount of colluvial cover from the more recent Wingate Sandstone and the steep angle of the exposures, resulting in rapid transport of any material exposed on the surface.

### Description

**Figure 5:**
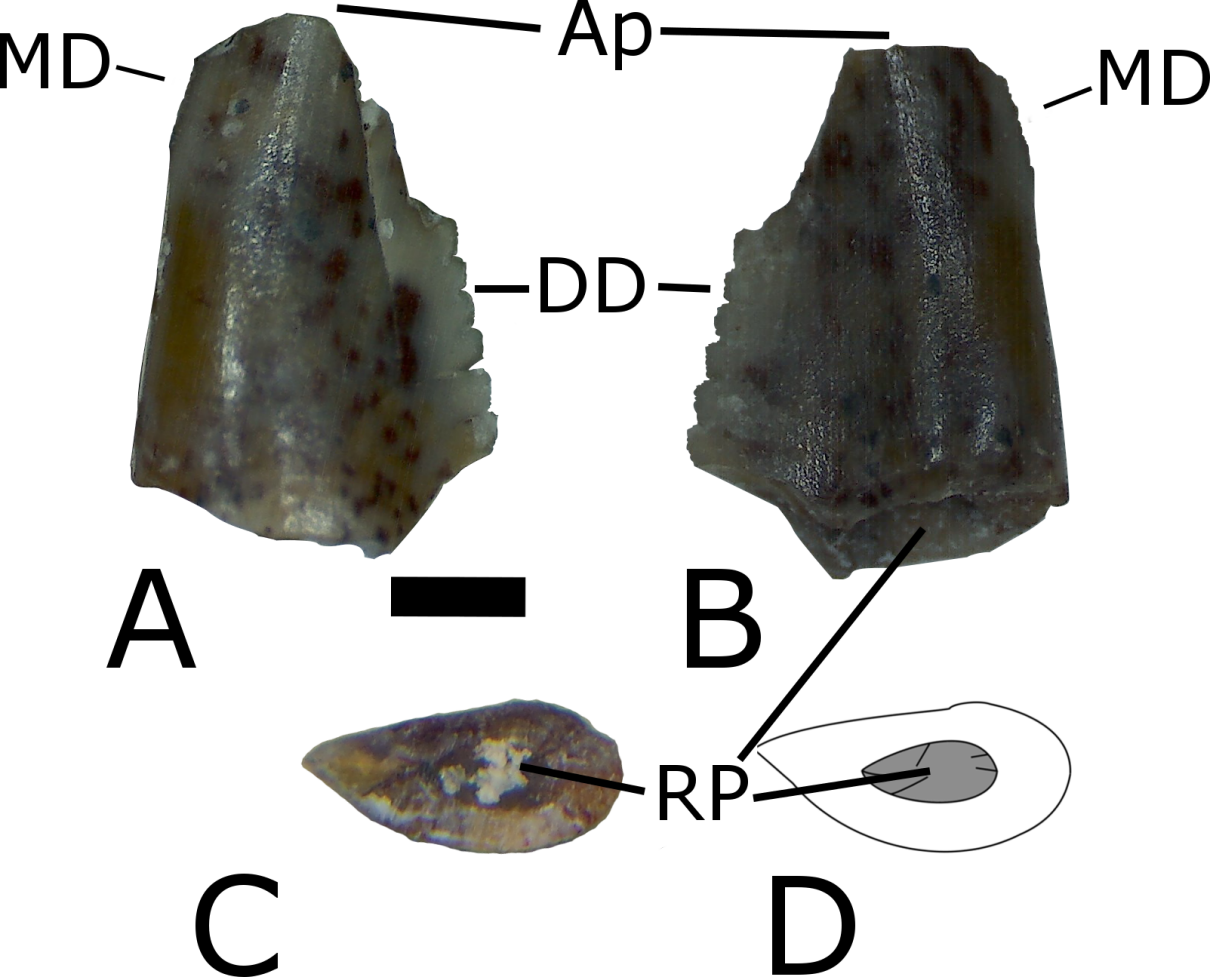
Multiple views of MNA V10666 (*Crosbysaurus* sp.). MNA V10666, *Crosbysaurus* sp., from MNA Locality 1725 in (A), presumed labial, (B), presumed lingual and (C), basal views. (D) Interpretive drawing in basal view showing resorption pit. Abbreviations: Ap, apex; DD, distal denticles; MD, mesial denticles; RP, resorption pit. Scale = 1 mm.

**Figure 6:**
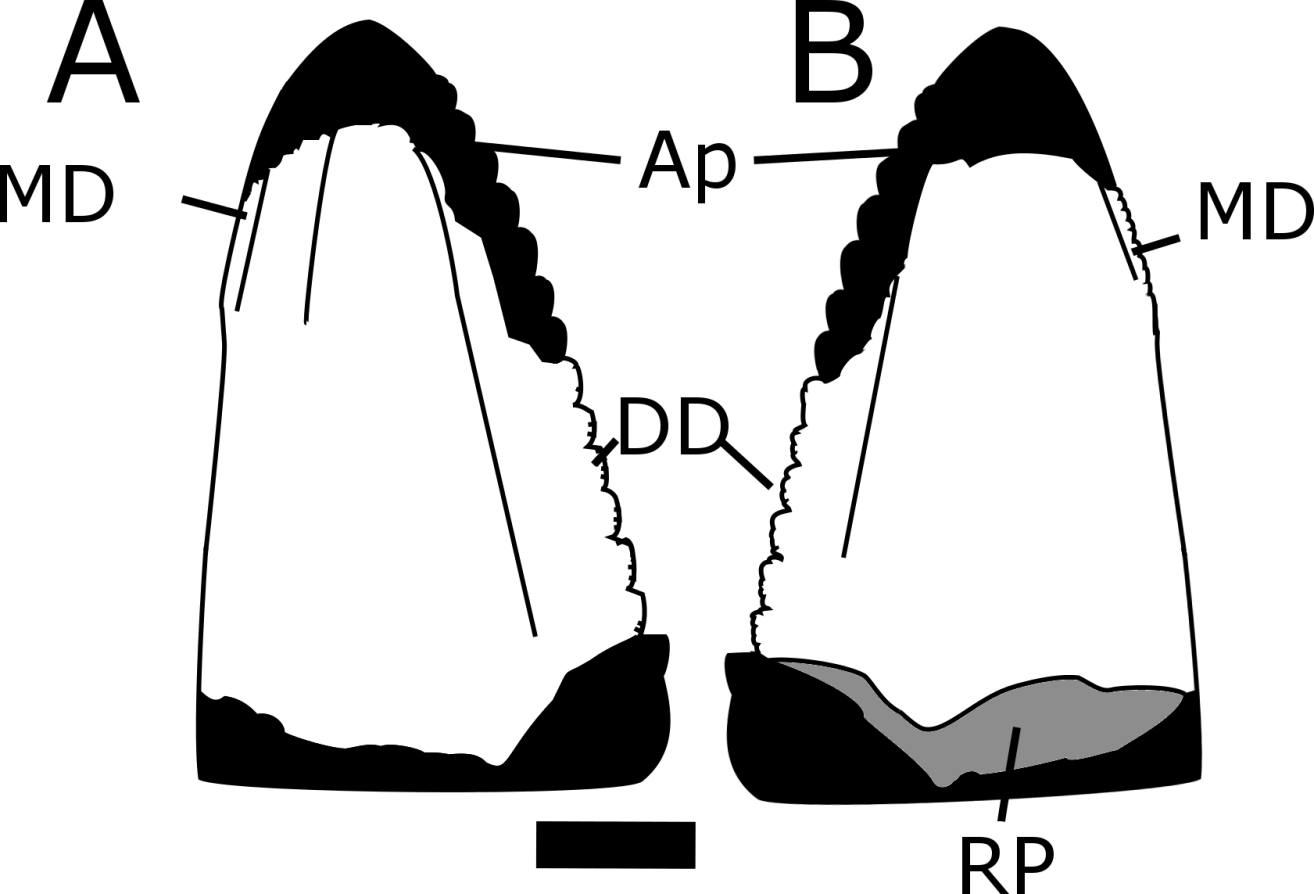
Line drawing of MNA V10666 (*Crosbysaurus* sp.). Interpretive line drawing of MNA V10666, *Crosbysaurus* sp., from MNA Locality 1725 showing the outline of the tooth if complete and the surface anatomy of the preserved crown. (A) presumed labial (B) presumed lingual views. Abbreviations: Ap, apex; DD, distal denticles; MD, mesial denticles; RP, resorption pit. Scale = 1 mm.

**Figure 7:**
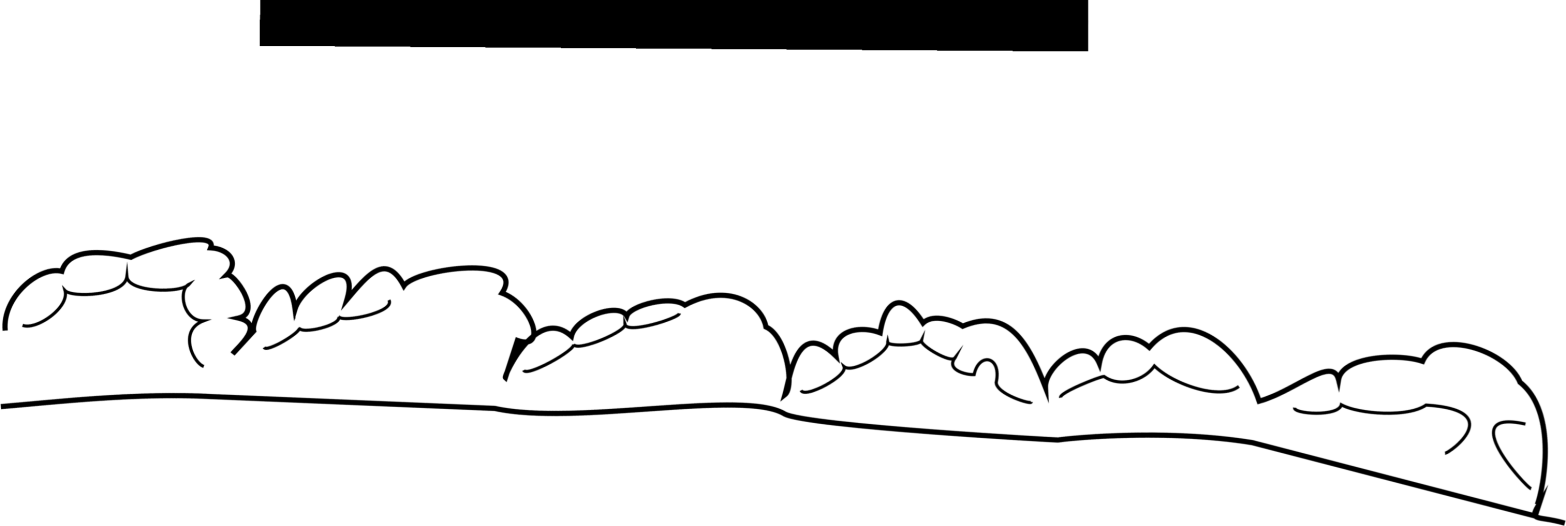
Line drawing of MNA V10666 (*Crosbysaurus sp.*) distal denticles Line drawing of the distal denticles of MNA V10666, *Crosbysaurus sp.*, from MNA Locality 1725. Apex is to the right, the base is to the left. Scale = 1 mm.

**Figure 8:**
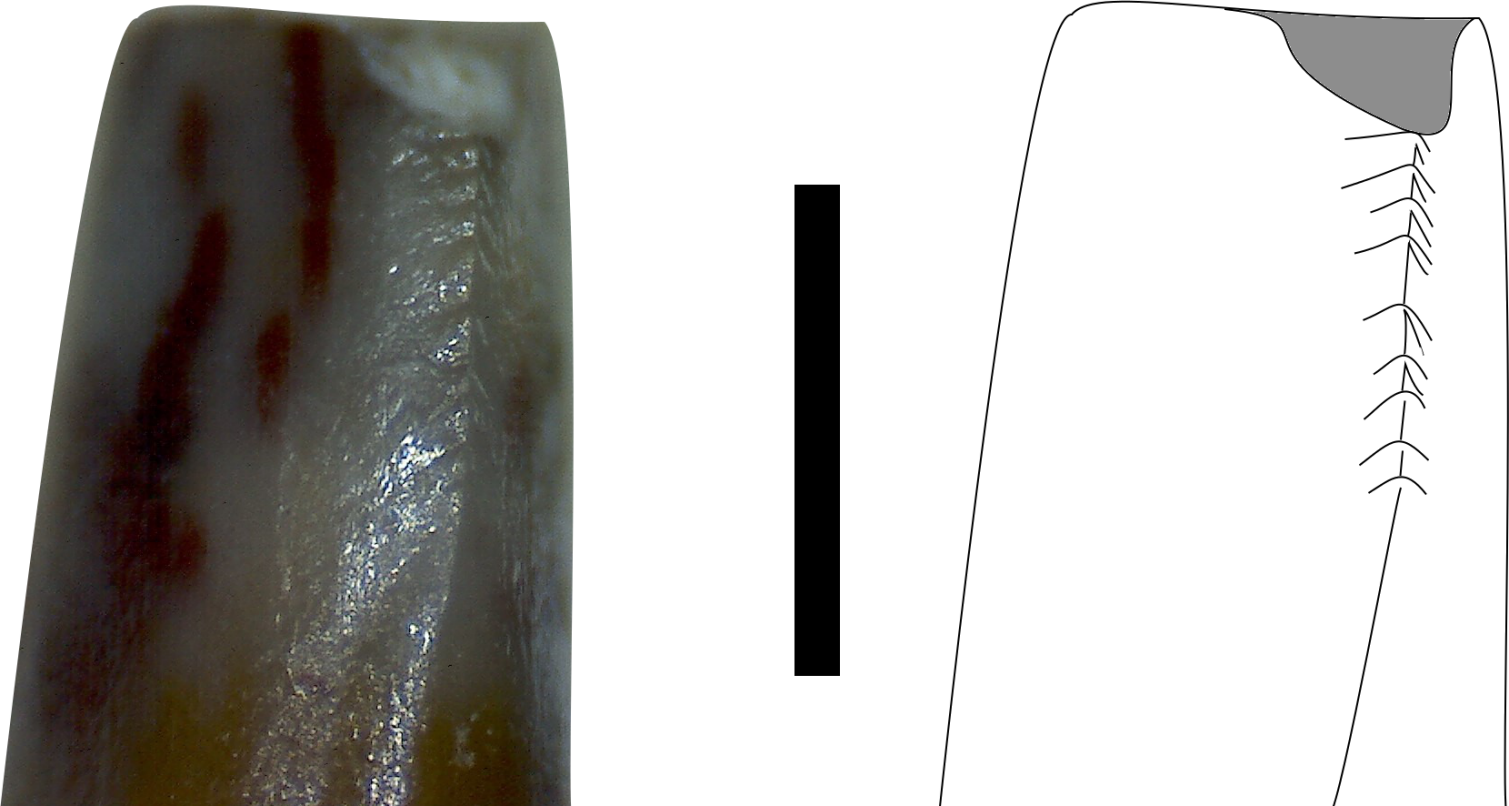
Mesial carina in mesial view of MNA V10666 (*Crosbysaurus* sp.) Photograph and interpretive line drawing of the mesial carina of MNA V10666, *Crosbysaurus* sp. The apex of the tooth is at the top of the image. Scale bar = 1 mm.

MNA V10666 (Figs. 5–8) is a single, nearly complete shed tooth crown. Since *Crosbysaurus* is known only from isolated dental material it is not possible to confidently assign a tooth row position to the MNA V10666. A Heckert (pers. comm., 2014) suggested to the first author that this tooth may be from the premaxilla based on the relative robustness. Given that *Crosbysaurus* cranial material is unknown, this is not currently testable. The tooth itself is labiolingually compressed and tapers mesiodistally towards the apex. There is an obvious resorption pit at the base of the tooth and the tip is taphonomically worn down (Fig. 5). These data suggest that MNA V10666 is a shed tooth crown. A reconstructed outline of the tooth is shown in Fig. 6.

The crown height is 3.7 mm from the base to the apically-most preserved point and 3 mm wide mesiodistally at the base. It is curved along the mesial margin and acute along the distal margin at the base of the crown. Labiolingually the tooth measures 1 mm (Fig. 5). The enamel color is a light tan to mottled brown, typical of many of the teeth from The Hills Have Teeth locality. The preserved distal edge of the tooth is straight and has six equally spaced denticles. The basalmost denticle is approximately 0.3 mm in apicobasal height. The apical-most denticle is 0.2 mm in height. Above the apical-most denticle there is a thin ridge of enamel. Since the tooth has been worn and was shed during life, it is damaged at the mesial and distal surfaces near the apex. This precludes determining if additional denticles may have been present higher on the distal side. This is not possible to evaluate at this time due to the premortem and postmortem wear of the tooth. The preserved posterior denticles possess smaller accessory serrations. Most of these are worn but one denticle preserves four accessory denticles on the basal edge and three on the apical edge (see Fig. 7).

The figured mesial edge of the tooth possesses a ridge that is expanded 2 mm from the base of the crown, approximately to the level of the last distal denticle. Very fine (<0.1mm) denticles cover the mesial edge of this ridge that extends for 1 mm. Below this ridge the crown’s enamel is intact and does not possess denticles, indicating this is the natural termination of this ridge (Fig. 8). The mesial denticles do not appear to possess accessory denticles, unlike what has been reported from other specimens of *Crosbysaurus* (Heckert, 2004).

## Discussion

### Taxonomic affinities

MNA V10666 differs from most described Triassic teeth with subdivided denticles assigned to herbivorous taxa in several features. It differs from the teeth of *Revueltosaurus*, the most commonly reported leaf-shaped-tooth in the Late Triassic of North America (Hunt, 1989; Heckert, 2002; Parker et al., 2005), by being labiolingually narrower. The teeth of *Revueltosaurus* are also mesiodistally broader compared to their apical-basal height. *Revueltosaurus* is now known from non-dental remains (Parker et al., 2005) and the tooth variation documented in the premaxilla, maxilla, and dentary do not match any teeth reported as *Crosbysaurus* (Irmis et al., 2007). This holds true for MNA V10666 as well; there appears to be no place in the dentition of *Revueltosaurus* for a tooth with the morphology of the described specimen.

*Krzyzanowskisaurus hunti* (Heckert, 2005) was originally named as a species of *Revueltosaurus* by Heckert (2002) based mainly on the presence of a cingulum on the tooth crown, separating it from the congeneric *R. callenderi*. After it was recognized that *R. callenderi* is in fact a pseudosuchian (Parker et al., 2005; Irmis et al., 2007), Heckert (2005) erected a new genus. Specimens formerly assigned to *R. hunti* were placed in this new genus, *Krzyzanowskisaurus*. At the time the change was done it was thought that *K. hunti,* represented a possible ornithischian (Heckert, 2005) but work by other authors (Irmis et al., 2007) challenged both its validity and its assignment to dinosauria. It is outside the scope of this paper to analyze the validity or affiliations of other Triassic tooth-based taxa. Regardless of the affinities of *K. hunti* it is clear that MNA V10666 does not possess a cingulum (Fig. 3). As such MNA V10666 cannot be referred to *Krzyzanowskisaurus*.

MNA V10666 differs from the Triassic tooth-based taxon *Tecovasaurus* in several ways. The teeth of *Tecovasaurus* tend to be much shorter and broader (Hunt & Lucas, 1994) as compared to MNA V10666 specifically, as well as to *Crosbysaurus* generally. Mesial denticles in *Tecovasaurus* tend to be large and coarse while being more numerous than those on the distal edge of the tooth (Heckert, 2004). In contrast, in MNA V10666 the preserved distal denticles are much coarser than those along the mesial edge (Fig. 6).

*Lucianosaurus wildi* (Hunt & Lucas, 1994) is another Late Triassic tooth-based taxon. Irmis et al. (2007) suggest it is an archosauriform though it was originally described as an ornithischian dinosaur. The holotype and referred specimen of *L. wildi* are dissimilar to MNA V10666 in that they possess finer denticles on both their mesial and distal edges. In addition, the tooth crowns of *L. wildi* are mesiodistally deeper than what is seen in MNA V10666. MNA V10666 is further differentiated by the steep mesial and distal edges that are nearly straight, compared to the low-angle convex edges of *L. wildi*.

The teeth of an unnamed taxon from the Owl Rock Member of the Chinle Formation of Arizona was described and figured by Butler, Porro & Heckert (2006). MNA V10666 differs from this unnamed taxon in several ways. The distal denticles of MNA V10666 are subdivided into multiple accessory denticles, unlike the simple cleft of the unnamed taxon. The mesial denticles of MNA V10666 are much smaller, not subdivided, and do not run to the base of the crown. The unnamed taxon is also much more symmetrical mesiodistally than MNA V10666.

*Protecovasaurus lucasi* (Heckert, 2004) differs from MNA V10666 because in the former the mesial and distal denticles are roughly equivalent in size and number. Indeed, no teeth reported for *Protecovasaurus* match the morphology seen in MNA V10666. Since non-dental fossils are not known for this or any other of the previously supposed ornithischians from the Triassic of North America, it is not possible to rule out positional or ontonogenic variation accounting for the morphological distance between MNA V10666 and these taxa. Given the homodonty present in most basal archosaurs and archosauriformes, it is unlikely that MNA V10666 represents any of the tooth-based taxa reported from the Late Triassic of North America.

MNA V10666 closely matches the published illustrations and descriptions of *Crosbysaurus harrisae* (Heckert, 2004). The complex distal denticles with multiple accessory serrations are an autapomorphy of *Crosbysaurus* (Heckert, 2004: 67, 68). None the less, several differences exist between MNA V10666 and all other described specimens that warrant some discussion.

Teeth referred to *Crosbysaurus* by other workers fall into two morphotypes: labiolingually compressed and highly recurved or basally wide and moderately recurved (see Heckert, 2004: 67, 68). MNA V10666 falls into neither category. It is labiolingually compressed, especially compared to other *Crosbysaurus* teeth in the literature, but without being recurved. This tooth lacks the labial bulge found in other specimens referred to *Crosbysaurus*. The distal denticles bear fewer accessory serrations than any other teeth referred to *Crosbysaurus*. The mesial denticles are much smaller, not compound, and are not found along the complete length of this tooth.

It is tempting to think that these differences may be systematically significant. However, we refrain from using these differences to taxonomically segregate MNA V10666 from other *Crosbysaurus* specimens for several reasons. The sample size from Utah is low (*n* = 1) and individual variation within this taxon has not been quantified. In addition, we lack any other body fossil remains from *Crosbysaurus* and it is currently unknown the dental variation along the tooth row, if present. Coupled with the taxonomic and systematic problems associated with *Revueltosaurus* (Hunt, 1989; Hunt & Lucas, 1994; Heckert, 2002; Parker et al., 2005; Heckert, 2005; Irmis et al., 2007; Heckert et al., 2012), a taxon whose relationships have been radically altered by the discovery of body fossils, we refrain from adding to the confusing plethora of tooth taxa known from the Late Triassic of North America.

Previous authors have suggested that *Crosbysaurus* is useful as a biostratigraphic index taxon of the St. Johnsian division of the Adamanian Land Vertebrate Faunachron assemblage (late Carnian-early Norian in age) (Heckert & Lucas, 2006). If these previous workers are correct, MNA V10666 may provide an important lower limit on the age of the Chinle Formation at Comb Ridge, an area that has received almost no paleontological and very little stratigraphic work in the past.

## Conclusions

The discovery of *Crosbysaurus* from the Chinle Formation of southeastern Utah extends the geographic range of this taxon northward by approximately 250 km. *Crosbysaurus* was apparently a rare but widespread taxon during the Chinle Formation deposition times. The single tooth crown recovered from Utah (MNA V10666) bears unique morphological characteristics that separate it from other described specimens of *Crosbysaurus,* as well as from other contemporaneous herbivorous archosaurs such as *Revueltosaurus callenderi*. These characters may represent various tooth positions within the jaw of *Crosbysaurus*, variation between individuals, or taxonomic differences. The sample size and preservation of known specimens of *Crosbysaurus* does not allow us to discriminate between these sources of variation at this time, so we refrain from making any statements about the primary cause. It is hoped that future work by Mission Heights’ field crews can help to better clarify the stratigraphic and taxonomic relationships of this enigmatic archosauriform.

## Abbreviations

MHPRO: Mission Heights Preparatory High School, Casa Grande, Arizona
MNA: Museum of Northern Arizona, Flagstaff, Arizona
NMMNH: New Mexico Museum of Natural History, Albuquerque, New Mexico

## Additional Information and Declarations

### Data Deposition

The following information was supplied regarding the deposition of related data: The poster describing the fieldwork and associated paleontology program has been archived on FigShare following its presentation in November 2014 with the following DOI: http://dx.doi.org/10.6084/m9.figshare.1222853.
